# Hedgehog Signalling Pathway and Its Role in Shaping the Architecture of Intestinal Epithelium

**DOI:** 10.3390/ijms252212007

**Published:** 2024-11-08

**Authors:** Adrianna Konopka, Kamil Gawin, Marcin Barszcz

**Affiliations:** 1 Laboratory of Analysis of Gastrointestinal Tract Protective Barrier, Department of Animal Nutrition, The Kielanowski Institute of Animal Physiology and Nutrition, Polish Academy of Sciences, Instytucka 3, 05-110 Jabłonna, Poland; m.barszcz@ifzz.pl; 2 Department of Animal Nutrition, The Kielanowski Institute of Animal Physiology and Nutrition, Polish Academy of Sciences, Instytucka 3, 05-110 Jabłonna, Poland; k.gawin@ifzz.pl

**Keywords:** Indian hedgehog, Sonic hedgehog, morphogen, intestine, villous, crypt, mesenchyme, proliferation, nutrition

## Abstract

The hedgehog (Hh) signalling pathway plays a key role in both embryonic and postnatal development of the intestine and is responsible for gut homeostasis. It regulates stem cell renewal, formation of the villous–crypt axis, differentiation of goblet and Paneth cells, the cell cycle, apoptosis, development of gut innervation, and lipid metabolism. Ligands of the Hh pathway, i.e., Indian hedgehog (Ihh) and Sonic hedgehog (Shh), are expressed by superficial enterocytes but act in the mesenchyme, where they are bound by a Patched receptor localised on myofibroblasts and smooth muscle cells. This activates a cascade leading to the transcription of target genes, including those encoding G1/S-specific cyclin-D2 and -E1, B-cell lymphoma 2, fibroblast growth factor 4, and bone morphogenetic protein 4. The Hh pathway is tightly connected to Wnt signalling. Ihh is the major ligand in the Hh pathway. Its activation inhibits proliferation, while its blocking induces hyperproliferation and triggers a wound-healing response. Thus, Ihh is a negative feedback regulator of cell proliferation. There are data indicating that diet composition may affect the expression of the Hh pathway genes and proteins, which in turn, induces changes in mucosal architecture. This was shown for fat, vitamin A, haem, berberine, and ovotransferrin. The Hh signalling is also affected by the intestinal microbiota, which affects the intestinal barrier integrity. This review highlights the critical importance of the Hh pathway in shaping the intestinal mucosa and summarises the results obtained so far in research on the effect of dietary constituents on the activity of this pathway.

## 1. Introduction

The development of the animal gastrointestinal tract has been the subject of research for many years due to its importance for nutrient utilisation, health, and economic outcomes of production. This process includes morphogenesis and cytodifferentiation occurring in the prenatal period as well as postnatal modifications induced by nutrients and mediated by endocrine and nervous systems [1]. In mammals, bioactive substances of colostrum and milk play a major role in the early postnatal development of the gastrointestinal tract, affecting the apoptosis-to-mitosis ratio and tissue rebuilding. Later in life, changes in diet composition and intestinal microbiota activity considerably affect the architecture of the intestinal epithelium [1]. The effects of nutritional factors on the morphological parameters of the gut have been extensively studied in pigs [2,3,4,5,6,7,8], poultry [9,10,11,12,13], laboratory rodents [14,15,16,17,18,19], and fish [20,21,22]. The histological parameters that are most often measured are villous height and crypt depth. Villi epithelium is covered by enterocytes responsible for absorption and secretions. These cells constitute 94% of the epithelial cells. The remaining cells are goblet (5%) and enteroendocrine cells (1%) producing mucus and local hormones, respectively. Intestinal crypts contain stem cells with a high rate of mitotic divisions, which serve as a replication centre. At the bottom of the small intestinal crypts, Paneth cells playing a role in host defence, mucosa development, and as a zinc reservoir are situated [23]. Thus, measuring villous height and crypt depth provides information on cell proliferation, mucosa function, and gut health. Changes in these parameters are induced by hormones, cytokines, cytotoxic agents, and local concentrations of trophic and growth factors [1].

Many pathways are involved in shaping the intestinal mucosa architecture, although one of them, the hedgehog (Hh) signalling pathway, seems to be of particular importance. It is a highly conserved pathway that transmits the signal from the cell surface to the nucleus. It was discovered in the fruit fly *Drosophila melanogaster* by Nüsslein-Volhard and Wieschaus [24] in the late 1970s as a gene whose mutation caused shortening of the segments and body and growth of a coat of setae on the underside of the larva’s body. Owing to the fact that mutated larva looked like a hedgehog, this locus affecting *Drosophila* segmentation was given the *hedgehog* name [24]. The Hh pathway plays a crucial role in embryogenesis because it regulates proliferation and differentiation of cells and tissue patterning [25]. In invertebrates, it is responsible for the proper segmentation and wing development, while in vertebrates, the pathway controls left–right asymmetry and proper formation of many tissues and organs, including the intestine [25]. In adults, this signalling pathway is involved in the maintenance of somatic stem cells and pluripotent cells, tissue repair, and regeneration. Impaired Hh signalling may contribute to many diseases, e.g., colon cancer [25,26].

In *Drosophila*, there is one *hh* gene, while in vertebrates, *Indian hedgehog* (*Ihh*), *Sonic hedgehog* (*Shh*), and *Desert hedgehog* genes were identified. The first one was named after a fictional hero of Sega’s video game “Sonic the Hedgehog”, while the others were named after real hedgehog species. Proteins encoded by these genes are mitogens, morphogens, and differentiation factors acting by short- or long-range signalling [27]. The morphogen model was proposed by Wolpert in the 1960s. The morphogen concentration gradient informs about the cell’s position, determining different cellular phenotypes that depend on the distance from the source of the signal. In a cell exposed to a morphogen, the thresholds of concentration determine which groups of target genes are expressed. Hh proteins are insoluble in water; however, these molecules are modified by lipids, which allows them to penetrate cells through an active mechanism [28]. Thanks to these properties, the concentration gradient of Hh proteins influences cell differentiation in the embryo. The Hh signalling pathway is closely linked to other signalling pathways that are involved in morphogenesis, organogenesis, and stem cell renewal in adults. These are the transforming growth factor-β/bone morphogenetic protein (TGF-β/BMP), Wnt/β-catenin, Notch, and fibroblast growth factor (FGF) pathways [25].

The aim of this review was to collect information on the Hh signalling pathway’s role in shaping the architecture of the intestinal mucosa with an emphasis on the effect of dietary factors on Hh signal transduction.

## 2. Hedgehog Protein Maturation

The maturation of Hh proteins is a three-step process. In the first step, the N-terminal signal sequence is removed from the ~45 kDa inactive precursor polypeptide encoded by Hh genes. Then, in the endoplasmic reticulum, it undergoes an autocatalytic cleavage between glycine and cysteine residues that results in the release of the N-terminal Hh signalling domain, which is covalently coupled to cholesterol at the C-terminus [29]. This results in formation of a ~19 kDa segment of all known signalling activities. In the third step, occurring at the plasma membrane, a membrane-bound O-acetyltransferase in *Drosophila* [30] or Hh acyltransferase in vertebrates catalyses palmitoylation of cysteine at the N-terminus. Then, a spontaneous S–N–acyl shift occurs and the stable amide bond is formed. This modification gives a signalling protein with full activity [25,31]. The release of mature, cholesterol-anchored Hh proteins from cells is regulated by a Dispatched protein containing a sterol-sensing domain (SSD) [32].

## 3. Receptors That Bind Hh and Modulate Its Signals

The Hh protein binds to Patched (Ptch), which is a 12-pass transmembrane receptor with two large hydrophobic extracellular loops that mediate Hh binding via an interaction of cholesterol with SSD [26]. There are two homologs in vertebrates: *Ptch1* [33] and *Ptch2* [34]. *Ptch1* is a transcriptional target of Hh signalling expressed in mesenchymal cells, it indicates the activated pathway and functions in a negative feedback loop [26]. Similarly, *Ptch2* is also an indicator of the activated pathway but it is expressed in epithelial cells of skin and testicles [26]. Ptch catalytically suppresses the activity of smoothened protein (Smo) [35], which is a co-receptor in the Hh signalling pathway. Smo is a positive regulator of the pathway, constitutively active when Ptch is absent, and promotes the activation of other components of the pathway [26]. The inhibitory effect of Ptch on Smo disappears upon binding to Hh protein. In *Drosophila*, the cytoplasm contains an inactive form of Smo, which accumulates on the surface of the cell after binding of the Hh ligand by Ptch [36]. In vertebrates, the inactive form of Smo occurs in the intracellular vesicles and cell membranes [37].

Another receptor that binds Hh proteins and participates in the signalling pathway is the Hh-interacting protein (Hhip). This is a target gene of this pathway that serves as its negative regulator [38] and competes with the Ptch receptor for binding Hh proteins [25]. In contrast, cell adhesion molecule-related/downregulated by oncogenes (Cdo) and brother of Cdo (Boc), found in vertebrates, facilitate binding and positively regulate the Hh signalling [39,40]. Boc plays the role of the Hh receptor on commissural axons, and both proteins are probably involved as receptors in the myogenesis regulation through the Hh signalling [41].

## 4. Intracellular Hh Signalling

The signalling cascade of the Hh pathways culminates in a change in equilibrium between activator and repressor forms of glioma-associated oncogene (Gli), which is the final target [26]. Glioblastoma transcription factors are zinc finger proteins highly conserved from *Drosophila* to vertebrates. There are three homologues in vertebrates: Gli1, Gli2, and Gli3 [42]. Gli1 is an activator of transcription [43] and indicates an activated Hh signalling pathway. It is one of the target genes of this pathway and provides positive feedback [26,44]. Gli2 mainly acts as a transcriptional activator but it may have weak repressive activity. This indicates that a small part of Gli2 may undergo proteolytic processing to form a transcriptional repressor. In mouse mutants lacking the *Gli3* gene, Gli2 may exhibit repressor activity and simultaneously function as a transcription activator. Proteolytic processing of Gli2 is very low relative to Gli3 [45]. The main effect of Gli3 is its repressor activity as most of the Gli3 protein (Gli3-190) is proteolytically processed to form the transcriptional repressor Gli3-83 in the absence of Shh signalling. When the Shh pathway is active, the Gli3 processing is blocked. Gli3 may also act as a weak activator of transcription due to incomplete proteolytic processing [45,46].

The main molecules of the Hh signalling pathway are clustered in the primary cilia, which can be found on almost every eukaryotic cell and participate in receiving signals from the extracellular environment [47]. These organelles are tail-like projections consisting of microtubules emanating from the cell surface. They are crucial for vertebrate development and human genetic disease and are specialised in Hh signal transduction [48].

When there are no Hh proteins, Ptch is found at the primary cilium base near the basal body [36] and blocks the formation of the active form of the Smo protein. Without the active Smo, full-length Gli, bound with a protein called suppressor of fused (Sufu), undergoes phosphorylation by protein kinase A, glycogen synthase kinase-3, and casein kinase 1. Then, it is proteolytically cleaved into a truncated Gli repressor or completely degraded (Gli2). The repressor goes into the nucleus, binds to promoters of Hh target genes, and prevents their transcription [26].

The signalling cascade becomes active after the binding of Hh proteins to Ptch. The complex of Hh and Ptch moves from the primary cilium to the cytoplasm and undergoes degradation in lysosomes. The translocation and degradation of the Hh-Ptch complex allows for the generation of the active form of Smo, which moves to the tip of the cilium, and for further signal transmission via a complex of cytoplasmic proteins composed of kinesin, Sufu, and full-length Gli. Sufu acts between Smo and Gli proteins and directly binds to Gli. Active Smo causes a movement of Sufu-Gli complex to the cilium. Then, the complex rapidly dissociates and the Gli activator is released. After that, it moves to the nucleus and switches on target gene expression [25,26].

Besides the abovementioned *Ptch1*, *Ptch2*, *Hhip*, and *Gli1*, the list of Hh signalling target genes includes the following: *G1/S-specific cyclin-D2* and *G1/S-specific cyclin-E1*; *MYCN proto-oncogene*; *B-cell lymphoma 2*; *FGF4*; *ATP binding cassette subfamily G member 2*; *vascular endothelial growth factor A*; *paired box 6*, *7*, and *9*; *jagged canonical Notch ligand 1*; and *forkhead box M1*. Of particular interest for shaping the intestinal mucosa architecture are genes encoding cell cycle regulators (cyclins), apoptosis regulator (B-cell lymphoma 2), protein engaged in cell proliferation and tumourigenesis (MYCN proto-oncogene), and mitogen involved in embryonic development, morphogenesis, and tissue repair (*FGF4*) [26].

## 5. The Hh Pathway in the Digestive Tract

Stem cell differentiation in the gastrointestinal tract is regulated along the vertical axis of the intestine and the longitudinal axis (oesophagus, stomach, small intestine, and colon), which determines the phenotype of epithelial cells produced by a specific stem cell. Morphogens are involved in the spatial regulation of cell differentiation during morphogenesis and play a key role in maintaining tissue homeostasis in adults along the two axes of the gastrointestinal tract. This also explains why mutations in morphogenetic pathways, such as the Wnt and BMP, can initiate carcinogenesis [49].

The adult epithelium of the small intestine is formed after completion of crypt formation in the third week after birth. Stem cells, which are situated just above the crypt base, produce enterocytes, goblet cells, and enteroendocrine cells, which migrate upwards towards the villous, and Paneth cells, which move downwards and populate the bottom of the crypts [50]. Hh proteins secreted by epithelial cells are responsible for the formation of crypts in the intestines [51].

There is very little data in the literature regarding the role of Hh signalling in the small intestine of adults. Low expression of the *Shh* gene was found in the human small intestine above the Paneth cells and in the colon but the level of protein expression was so low that it could not be detected by immunohistochemical staining [52]. *Shh* expression is the highest in the ileum and the proximal part of the colon [53] and may play a specific role in the homeostasis of the distal ileum in adults. In *Shh* knockout mice (*Shh*^ΔIEC^), it was observed that the length of the small intestine was shorter in comparison with controls and there was no compensatory change in the expression of *Ihh*. In these mice, no significant histological changes were observed in most sections of the intestine, while in the ileum, a 1.2 times shorter crypt/villous axis was shown compared to the control animals. No changes in the number of apoptotic cells were observed in the proximal and distal segments of the intestine. Also, there were no changes in the jejunum but in the ileum, a 1.3-fold decrease in the number of proliferating cells was noted. These findings indicated that the reduction in the crypt/villous axis in the ileum may depend on a deregulation of the proliferation of epithelial cells. Moreover, the ileum of the knockout mice was characterised by a decrease (1.2-fold) in the number of goblet cells producing acidic mucins, a defect in mucin fucosylation, and by a decrease (1.3-fold) in Paneth cell count [54]. The number of Paneth cell granules did not differ between the control and *Shh*^ΔIEC^ group but the granules were significantly smaller in knockout animals. Moreover, the expression of defensin 4 was significantly decreased, while that of inositol-requiring 1 alpha—a marker of endoplasmic reticulum stress—was higher in the ileum of *Shh*^ΔIEC^ mice. This part of the small intestine was also characterised by an autophagy impairment, demonstrated by accumulation of p62 protein and a decreased ratio of the lipidated (membrane-bound) to non-lipidated (cytosolic) form of microtubule-associated protein 1 light chain 3 beta [54].

Paneth cells reside at the crypt bottom and take part in intestinal defence by secreting bactericidal peptides, i.e., α-defensins, lysozyme, and immunoglobulin A. They are also involved in the processes of angiogenesis and intestinal cancer as well as inflammatory bowel diseases. These cells express the CD1 marker and synthesise many inflammatory mediators, such as granulocyte-macrophage colony-stimulating factor, tumour necrosis factor-α, prostaglandin E2, and Fas ligand. Owing to these properties, these cells may coordinate an immune response, both innate and adaptive [55]. Differentiation of Paneth cells requires the activity of peroxisome proliferator-activated receptor-β (PPARβ), which enables the differentiation of their precursors by inhibiting the transduction of the Hh signal [55].

PPARβ is a transcriptional factor that belongs to the superfamily of nuclear receptors involved in metabolic homeostasis and activated by binding to a ligand, i.e., most fatty acids and their derivatives—prostaglandins and leukotrienes [55]. PPARβ is expressed in all sections of the small intestine, and is found mainly in epithelial cells with an increasing gradient from the top to the bottom of the villi. The highest PPARβ expression is found at the crypt bottom. In *PPARβ*^−/−^ mice, *Ihh* expression was 3-fold higher than in wild-type mice. The increase in *Ihh* resulted in the upregulation of *BMP4*, which is a signalling target of the Hh pathway [56]. In wild-type mice, the activation of PPARβ by oral administration of its synthetic selective agonist resulted in a decrease in *Ihh* expression along with a concurrent downregulation of *BMP4*. This suggests that PPARβ negatively controls the Ihh signal in the small intestine [55]. An experiment conducted on wild-type and *PPARβ*^−/−^ mice treated with cyclopamine, an inhibitor of the Hh pathway [57], showed an increased number of Paneth cells by approximately 30%. This confirmed the association between PPARβ-dependent Hh regulation and reduced differentiation of Paneth cells in *PPARβ*^−/−^ mice [55].

In research on transgenic mice concerning the effects of the abolition of Hh signals in the small intestine after birth, it was observed that the epithelium was flattened and the remodelling and development of villi were impaired. It was also noted that mesenchymal changes (expansion of smooth muscle progenitors and inappropriate localisation of subepithelial myofibroblasts) secondarily increased the proliferation of epithelial cells and activity of target genes of TCF4/β-catenin pathway. Thus, the Hh pathway shapes the crypt/villous axis in the neonatal small intestine through paracrine signalling, from epithelium to subepithelial myofibroblasts and smooth muscles expressing *Ptch1* [51].

Studies on wild-type mice showed that blocking the Hh pathway with anti-Hh 5E1 monoclonal antibodies caused the death of all animals three weeks after birth. Mice had emaciation with severe diarrhoea, major histological abnormalities, disorganised intestinal villi protruding into the intestinal lumen, and crypt hyperplasia with a 73% increase in cell proliferation in comparison with controls. There was significant alveolar vacuolisation in enterocytes, mainly in the ileum and caecum, and accumulation of neutral lipids in the vacuoles. In the stool of these mice, there were numerous microscopic fat droplets, while in the blood, concentrations of total cholesterol, apolipoprotein A-IV, and high-density lipoproteins were lower [58].

In the duodenum of adult mice, the expression of *Ihh* was found in differentiated epithelial cells on the villi [59]. Loss of *Ihh* expression resulted in a substantial reduction in expression of Hh target genes, i.e., *Gli1*, *Hhip*, *Ptch1*, and *Ptch2,* and initiation of the epithelial wound-healing response. The rate of crypt fissioning and cell proliferation were increased to replace the lost crypts. Deletion of *Ihh* increased Wnt signalling but BMP signalling from the epithelium of the villi and activin signalling from crypts were lost. The lack of Hh signalling caused a migration of fibroblasts and macrophages to the villous core, which are principal cells participating in wound healing. These changes were accompanied by an increased signalling of TGF-β in the mesenchyme and deposition of proteins of the extracellular matrix. This research demonstrated also that extended loss of Ihh signalling led to progressive migration of macrophages, neutrophils, and T lymphocytes to the crypt area and the development of chronic enteritis and intestinal fibrosis. In mutant mice, villous atrophy in the small intestine gave it an appearance of the colon mucosa and resembled pathological changes occurring in patients with coeliac disease. The difference was that in *Ihh*-deficient mice, there were no intraepithelial lymphocytes, while coeliac disease is characterised by their accumulation. Therefore, it was suggested that in people suffering from the disease, the immune response to gluten may contribute to the loss of *Ihh* expression and, in turn, to villous atrophy [59].

In the small intestine of *Ihh*^−/−^ mice there is a 34% decrease in the thickness of the circular smooth muscle layer and in *Shh*^−/−^ mice there is a 21% reduction as compared to wild-type animals [60]. Abnormalities in the enteric nervous system were found in both *Shh* and *Ihh* knockout mice. *Shh*^−/−^ mice show a high number of neurons abnormally differentiated under the epithelium and into the villi. In normal epithelium, *Shh* inhibits the proliferation of neurons, and this activity is stopped after administration of cyclopamine [61]. *Shh* causes proliferation of neural crest cells through the inhibition of differentiation and modulation of their response to glial cell lineage-derived neurotrophic factor [62]. In *Ihh*^−/−^ mice, there are sections in the small intestine and dilated regions in the colon with a lack of neurons, which indicates that Ihh signalling is essential for the development of gut innervation [60].

**Figure 1:**
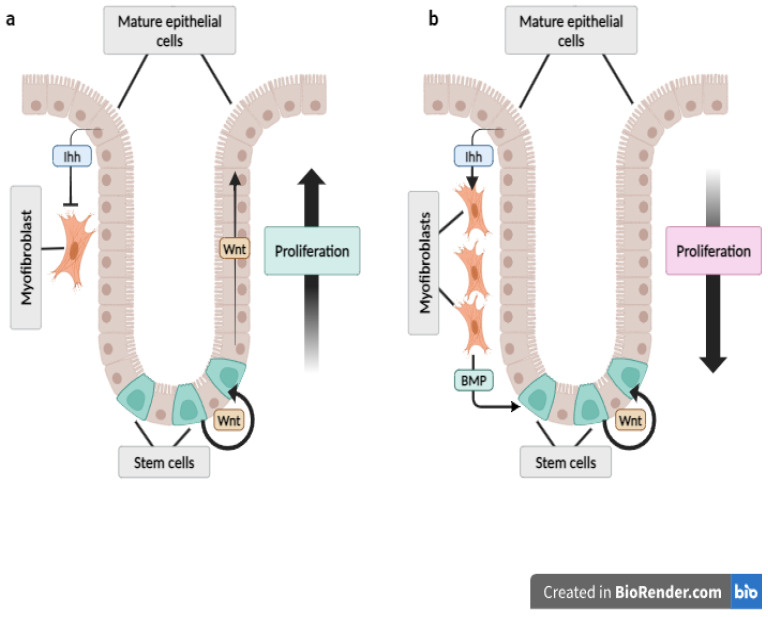
(**a**) Inhibition of the hedgehog signalling pathway leads to the increase in proliferation and upregulation of Wnt signalling. These events lead to crypt elongation. (**b**) Enhanced hedgehog signalling in the mesenchyme causes accumulation of myofibroblasts, reduction in Wnt signalling, and proliferating cell number at the base of the crypt via bone morphogenetic proteins. These events contribute to crypt shortening.

Homeostasis of the small intestine epithelium, including crypt and villous architecture, proliferation and differentiation of cells, and apoptosis, are regulated spatially and temporally by multiple signalling pathways [63]. Intestinal cell differentiation is in homeostatic balance dependent on a negative feedback loop. The proliferation and fate of intestinal precursor cells is regulated by Wnt signalling. Inhibition of the Hh pathway activity by cyclopamine resulted in increased Wnt signalling and proliferation of precursor cells, while enterocyte differentiation was impaired. *Ihh*^−/−^ mice were characterised by a lack of differentiation of proliferating cells and impaired formation of crypts. Such mice were unable to live [64]. The Hh signalling inhibition during the development of the intestine by Hhip led to the accumulation of proliferating cells, which in turn caused an upregulation of Wnt signalling and crypt elongation (Figure 1a) [51].

Activation of Hh signalling in the colon of adult mice by induced deletion of exons 8 and 9 of *Ptch1* led to constitutive signalling through Smo, inhibition of Wnt signalling, and a reduction in the number of precursor cells that were prematurely differentiated into enterocytes [65]. Wnt signalling is crucial for the specification of the fate of precursor cells. A feature of activated Wnt signalling is the accumulation of β-catenin in the nucleus of intestinal epithelial cells. In control mice, β-catenin accumulated in the cytoplasm of cells at the bottom of the crypts and nuclear positivity was found, while in the mutant mice, a loss of cytoplasmic accumulation and exclusion of β-catenin from the nucleus were observed [65]. The loss of Wnt signalling was confirmed by downregulation of the expression of three Wnt signalling targets, i.e., EPH receptor B2, EPH receptor B3, and the CD44 antigen in the colon of mice with the mutated *Ptch1* gene, which showed large areas with hypoplastic crypts [65]. This impairment likely resulted from reduced proliferation of epithelial cells rather than increased apoptosis. In wild-type mice, there were large numbers of round epithelial precursor cells in the colonic crypts, with typical features of undifferentiated cells, such as little cytoplasm or darkly stained and large nuclei. In the colon of mice with the *Ptch1* mutation, the precursor cells located at the crypt bottom were partially replaced by prevacuolated cells having a smaller nucleus of flattened shape and a large number of vacuole-like granules in the apical part of the cytoplasm [65]. These results indicated that improper Hh signalling leads to depletion of the compartment of precursor cells in crypts and premature development of colonocytes. In *Ptch1* mutant mice, there were no changes in villin expression, goblet cell number, and enteroendocrine cell number but myofibroblast accumulation was noted. However, increased expressions of carbonic anhydrase II and caudal-type homeobox 2, which are markers of differentiated enterocytes, were found [65]. Although villin is also a marker of enterocyte differentiation, the reasons for the lack of changes in its expression in *Ptch1* mutant mice, as compared to the wild-type animals, are unknown.

In the colon of rats, mature colonocytes express *Ihh* mRNA and protein, which regulate their differentiation and restrict Wnt signalling to the crypt base (Figure 1b). Hh signalling also restricts the expression of engrailed-1—which is a target gene of the Wnt pathway—and *BMP4* to the compartment of the precursor cells at the bottom of colon crypts. Immunohistochemistry showed that when the Hh pathway is inactivated, BMP4 is expressed by the epithelial cells on the entire length of the crypt [64]. Studies on *Ptch1* mutant mice confirmed that the main Hh ligand in the colon is Ihh. It is secreted by differentiated colonocytes to the mesenchyme. The upregulated Hh signalling in the mesenchymal cells secondarily increases the expression of BMPs in the epithelial cells and extends the signalling of epithelial BMP toward the crypt bottom (Figure 1b). In the colon of mutant mice, the expression of *BMP2*, *BMP4*, and *BMP7* increased. *BMP2* expression was restricted to differentiated colonocytes. In control mice, *BMP4* was expressed mainly in the epithelial cells, while in *Ptch1* mutants, mainly in the mesenchymal cells. *BMP7* was expressed at the higher parts of the crypts by the mesenchymal cells underlying the intercrypt epithelium [65].

## 6. Nutrition and Hh Signalling

Availability of nutrients determines the growth and development of organisms. Studies on fruit flies showed that the fat body, which is analogous to white adipose tissue and liver in mammals, plays an important role in linking nutrient availability to growth. In response to changes in nutrient availability, it releases factors regulating growth by modulation of systemic insulin signalling. The fat body also has storage functions and releases nutrients during starvation [66]. The availability of nutrients also controls the production of ecdysteroids in the prothoracic gland, thus affecting the developmental progression. These hormones are secreted in pulses that are responsible for the regulation of molting and initiation of pupariation [66]. In fruit flies, the intestine regulates the production of lipoprotein-modified Hh circulating in the body in response to the availability of nutrients. The fat body responds to circulating Hh, which controls the growth of larvae. By controlling ecdysteroid production, Hh regulates developmental timing. During starvation, Hh is also particularly important because it is necessary for the mobilisation of triacylglycerols from the fat body. This research showed that circulating Hh, produced by the intestine, acts as a hormone, which coordinates the reaction of different tissues to the availability of nutrients. Similar functions may be exerted by circulating Shh in mammals [66].

### 6.1. Fat

Dietary fat has a considerable impact on intestinal morphology. Feeding a diet with a low ratio of n-6 to n-3 polyunsaturated fatty acids reduced crypt depth in the duodenum of 17-day-old broilers and villi height in the jejunum of 43-day-old chickens [10]. In mice, a diet rich in α-linoleic acid as well as a high-fat diet rich in cholesterol increased mucosa thickness, villi height, and the epithelial cell number in the middle part of the small intestine. Both diets reduced the proportion of goblet and Paneth cells as well as the proliferation rate [18]. Studies on mice showed that Hh signalling may be involved in abnormalities in lipid absorption caused by diet [67]. Inactivation of Hh signalling by administration of anti-Hh monoclonal antibodies protected adult mice given a high-fat diet from weight gain. Such inactivation of Hh signalling was found to be effective also in the case of the genetic form of obesity, which was shown in studies on leptin-deficient mice fed a low-fat diet [67]. Inhibition of Hh signalling resulted in a delayed rate of triglyceride absorption, greater faecal output of total lipids and free fatty acids, and increased expression of apolipoprotein AIV in the intestine. There was also an increase in the expression of the *3-hydroxy-3-methylglutaryl-CoA synthase* gene in the epithelial cells of the intestine and decreased *Gli1* and *Ptch2* expressions in intestinal stromal cells [67].

Diets with a high content of fat are a significant hazard factor for colorectal cancer development and lipids are important for the deregulation of the Hh signalling pathway, which contributes to carcinogenesis [68]. The metabolism of lipids affects the transduction of the Shh signal because Smo inhibition by Ptch1 can be relieved by the oxysterols being derivatives of cholesterol. A high-fat diet may impair the activation of Hh signalling and the proliferation of cells, cause a reduction in the apoptosis rate, and intensify stem cell renewal. The cholesterol-induced aberrant Shh pathway signalling can be blocked by the activation of AMP-activated protein kinase [68].

### 6.2. Vitamin A

Inclusion of retinoic acid, an active metabolite of vitamin A, in a diet increased crypt depth and villous height in the duodenal–jejunal and ileal segments of rats after small bowel resection. In these segments, retinoic acid stimulated the proliferation of crypt cells and inhibited apoptosis. The study also showed that the small bowel resection decreased *Ihh* and *Shh* expression and that retinoic acid downregulated *Ptch1*, *Ptch2*, and *Gli1* and tended to decrease *BMP4* expressions [69]. These results indicate that retinoic acid treatment enhances adaptation of the small intestine after the surgery and that the response is related to the inhibition of the Hh signalling pathway activity [69].

### 6.3. Berberine

Another dietary constituent that affects mucosal architecture and the Hh signalling pathway is berberine, a benzylisoquinoline alkaloid present in many plants, e.g., *Berberis aristata*, *Berberis vulgaris*, *Coptis chinensis*, and *Hydrastis canadensis* [70]. Evidence showed that a high dose of this compound (1 g/kg diet) increased the height of the villous and tended to reduce duodenal crypt depth in 12-day-old chickens, and reduced crypt depth in the duodenum and jejunum of 21-day-old birds [71]. These data indicate that berberine enhances the integrity of the intestinal barrier [71]. Studies on weaned piglets fed a diet supplemented with 10 mg berberine/kg body weight showed increased villous height and decreased apoptosis in the jejunum [72], which confirmed the potential of this alkaloid to shape the mucosal architecture. Research on the use of berberine as an anticancer agent revealed that this compound decreases *Shh*, *Ptch1*, *Smo*, and *Gli1* expressions but increases the *Sufu* level in colorectal cancer tissue in mice in a concentration-dependent manner [70]. These results showed that berberine likely inhibits cancer growth by interrupting the paracrine Hh signalling cascade. As an Smo inhibitor, berberine binds to the same site as cyclopamine [73]. These data confirm that downregulation of the Hh signalling pathway may be the primary mechanism of the anticancer activity of berberine [70]. However, the relationship between intestinal morphology, Hh signalling, and berberine remains to be unraveled.

### 6.4. Ovotransferrin

Ovotransferrin is a glycoprotein from egg white with iron-binding properties [74]. Due to the antibacterial, antiviral, anti-inflammatory, antioxidant, and immunomodulatory effects [75], ovotransferrin has the potential to be used in nutraceutical and functional foods. Studies on mice revealed that this glycoprotein may also affect colon mucosa morphology and Hh signalling pathway activity. Administration of ovotransferrin via oral gavage (5 mg/mL in water) to three- and eight-week-old mice for two weeks increased crypt depth in the colon, which was attributed to greater abundance of *Akkermansia*—beneficial bacteria that enhanced the epithelial integrity [76]. In the study, ovotransferrin treatment also increased the goblet cell number in adult mice and downregulated the *Shh* expression in young mice [76].

### 6.5. Haem

Another dietary constituent known for its effect on gut morphology and Hh signalling pathway is dietary haem. It is an iron-porphyrin pigment found in red meat and an important risk factor of colon cancer development [15]. Rats receiving haem in a diet had increased cell proliferation rate and crypt depth in the colon and disrupted structure of the surface epithelium [15]. Further research showed that haem-induced stress at the surface epithelium and the resulting loss of feedback signals lead to hyperproliferation of cells in the colonic crypts. *Wnt inhibitory factor 1*, *Ihh*, and *BMP2* were the downregulated inhibitors of proliferation. *Ihh* inhibits proliferation by stimulating the secretion of BMPs, which are Wnt antagonists, from the lamina propria cells [77]. The obtained results agreed with the previous one, demonstrating that the activation of Ihh signalling decreases proliferation while its blocking causes hyperproliferation [64,65].

### 6.6. Intestinal Microbiota

Diet has a considerable impact on intestinal microbiota, which affects gut morphology and the development of the intestinal immune system [1,78]. The epithelial barrier of the intestine and epithelial cell renewal are regulated by the signalling pathways of pattern recognition receptors, and in particular by toll-like receptors (TLR). These receptors are used by intestinal epithelial cells to detect pathogen-associated molecular patterns of gut-dwelling microorganisms. Recent evidence indicated that the recognition of these patterns via TLR affects the intestinal barrier function and that TLR2 signalling connects microbial colonisation with the regulation of the Hh signalling pathway [79]. Ihh protein is secreted by differentiated enterocytes, while the expression of its mRNA is highest at the junction of the villous and the crypt. Studies on germ-free and conventional mice showed that the former had a higher Ihh protein expression in the small intestine and a strengthened intestinal barrier. This was associated with elevated levels of epithelial neuropilin-1, which is the type I transmembrane glycoprotein and a positive-feedback regulator of Hh signalling. This glycoprotein was found to be a critical element that increases the activity of the Hh signalling pathway, thereby improving the intestinal barrier [79]. Colonisation of the small intestinal epithelium by microbiota activates TLR2 signalling, which leads to a reduction in neuropilin-1 expression on the epithelial cell surface through a pathway of lysosomal degradation. Downregulation of neuropilin-1 suppresses the activity of the Hh signalling pathway, which weakens the intestinal barrier and increases gut permeability via a reduction in the expression of tight junction proteins. Microbiota-induced TLR2 activation suppresses also *BMP4* expression in the intravillous mesenchyme [79].

Another aspect of intestinal microbiota influence on the activity of the Hh signalling pathway is related to microbial production of folic acid. This vitamin is a methyl donor necessary for the one-carbon metabolism and DNA methylation [80] and *Bifidobacterium bifidum* and *Bifidobacterium longum* subsp. *infantis* were identified as a high-level folate-producing species among human gut commensals [81]. This modification of DNA structure is the primary epigenetic mechanism of regulation of gene expression. Hypermethylation of CpG islands in the gene promoter leads to gene silencing, while hypomethylation gives an opposite effect [80]. It was shown that several key genes of the Hh pathway (*Ptch1*, *Hhip*, *cyclin D2*, and *secreted frizzled-related protein 1*) have CpG islands in their promoters [82] and the demethylation of the *Shh* promoter leads to its overexpression [83]. Thus, microbial production of folic acid as well as supplementation of a diet with this vitamin may affect the Hh pathway and all aspects of gut physiology regulated by this signalling. In a similar manner, the expression of Hh ligands may be regulated by another vitamin of microbial origin, i.e., vitamin B12, which is also involved in the one-carbon metabolism and methylation of DNA [80] and is produced by *Lactobacillus reuteri* [81].

## 7. Summary

**Figure 2:**
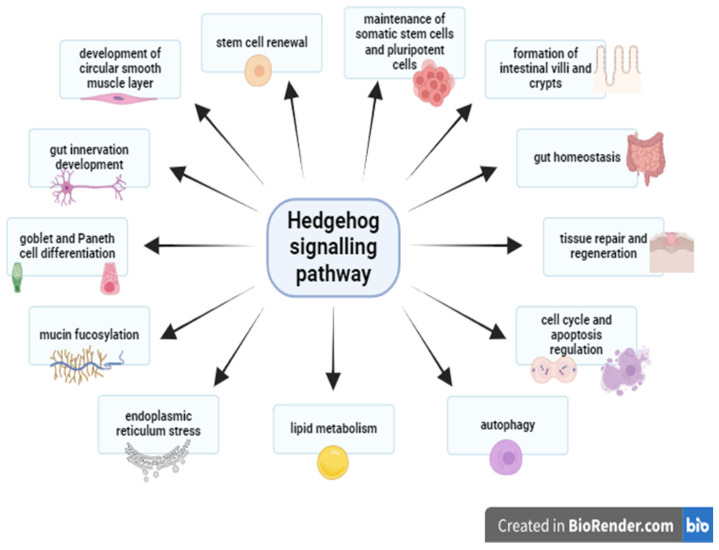
The summary of the role of the hedgehog signalling pathway in shaping the intestinal mucosa in adults.

The Hh signalling pathway is involved in the maintenance of somatic stem cells and pluripotent cells, renewal of stem cells, formation of the villous–crypt axis, differentiation of goblet and Paneth cells, mucin fucosylation, tissue repair, regulation of the cell cycle and apoptosis, development of the circular smooth muscle layer and gut nervous system, endoplasmic reticulum stress and autophagy, and lipid metabolism (Figure 2).

Hh ligands are expressed by terminally differentiated epithelial cells and secreted from the epithelium to the mesenchyme, where they are bound by Ptch receptors localised on myofibroblasts and smooth muscle cells. This binding reduces the inhibitory effect of Ptch on Smo and activates Gli transcription factors and the expression of target genes. By stimulating the expression of proteins from the BMP family, active Hh ligands inhibit Wnt signalling in a stem cell niche of intestinal crypts and limit uncontrolled proliferation and epithelial renewal. It is known that Ihh is the major morphogen in this pathway and its activation inhibits proliferation, while the loss of this signal induces hyperproliferation and triggers a wound-healing response. Thus, Ihh is a negative feedback regulator in the dynamic equilibrium between cell proliferation and cell loss and is a key signal indicating the integrity of the superficial epithelium. Both epithelial morphogens, Ihh and Shh, are involved in the normal development of the intestine but their role in the gut physiology of adults is still poorly recognised. As numerous studies have shown the effect of a diet on intestinal morphology, research on the role of diet composition in the regulation of the activity of the Hh signalling pathway seems to be of particular importance. This becomes even more important in light of recent evidence indicating that the intestinal microbiota, whose composition and activity strongly depend on diet, affects the Hh pathway and intestinal barrier function. Therefore, to provide better insight into the molecular nature of diet-induced changes in villous height or crypt depth, the Hh signalling pathway should be explored in nutritional experiments, which would considerably extend the existing knowledge in this field.
